# Constructing a Nomogram Model to Estimate the Risk of Ventilator-Associated Pneumonia for Elderly Patients in the Intensive Care Unit

**DOI:** 10.3390/arm92010010

**Published:** 2024-01-19

**Authors:** Wensi Gan, Zhihui Chen, Zhen Tao, Wenyuan Li

**Affiliations:** 1 Department of Infection Control, Wenzhou Hospital of Integrated Traditional Chinese and Western Medicine, Wenzhou 325001, China; 2 Department of Big Data in Health Science, School of Public Health, Zhejiang University, and Center for Clinical Big Data and Statistics, The Second Hospital Affiliated to Zhejiang University School of Medicine, Zhejiang University, Hangzhou 310058, China; 3 School of Public Health, Zhejiang University, Hangzhou 310058, China; 4 Department of Intensive Care Unit, Wenzhou Hospital of Integrated Traditional Chinese and Western Medicine, Wenzhou 325001, China

**Keywords:** nomogram, VAP, prediction model, infection prevention, ICU, elderly patients

## Abstract

**Highlights:**

**What are the main findings?**

- The predictors included in the final nomogram model were hypoproteinemia, long-term combined antibiotic use, intubation times, mechanical ventilation days, and tracheotomy/intubation.
- The area under the curve (AUC) was 0.937 (95% CI: 0.902–0.972) and 0.925 (0.867–0.982) in the training and validation datasets, respectively, suggesting that the model demonstrated effective discrimination. Our model also demonstrated strong concordance performance and clinical applicability.

**What is the implication of the main finding?**

- Using this nomogram model, clinicians can assess VAP risk in elderly ICU patients and identify those at high risk.
- External validation of the nomogram model in larger populations is still required.

**Abstract:**

Background: Ventilator-associated pneumonia (VAP) causes heavy losses in terms of finances, hospitalization, and death for elderly patients in the intensive care unit (ICU); however, the risk is difficult to evaluate due to a lack of reliable assessment tools. We aimed to create and validate a nomogram to estimate VAP risk to provide early intervention for high-risk patients. Methods: Between January 2016 and March 2021, 293 patients from a tertiary hospital in China were retrospectively reviewed as a training set. Another 84 patients were enrolled for model validation from April 2021 to February 2022. Least absolute shrinkage and selection operator (LASSO) regression and multivariable logistic regression analysis were employed to select predictors, and a nomogram model was constructed. The calibration, discrimination, and clinical utility of the nomogram were verified. Finally, a web-based online scoring system was created to make the model more practical. Results: The predictors were hypoproteinemia, long-term combined antibiotic use, intubation time, length of mechanical ventilation, and tracheotomy/intubation. The area under the curve (AUC) was 0.937 and 0.925 in the training and validation dataset, respectively, suggesting the model exhibited effective discrimination. The calibration curve demonstrated high consistency with the observed result and the estimated values. Decision curve analysis (DCA) demonstrated that the nomogram was clinically applicable. Conclusions: We have created a novel nomogram model that can be utilized to anticipate VAP risk in elderly ICU patients, which is helpful for healthcare professionals to detect patients at high risk early and adopt protective interventions.

## 1. Introduction

Ventilator-associated pneumonia (VAP) refers to pneumonia arising more than 48 h after tracheotomy or endotracheal intubation for mechanical ventilation. VAP also includes pneumonia that occurs within 48 h after the removal of mechanical ventilation and extubation. In intensive care units (ICUs), mechanical ventilation is commonly used to save patients’ lives. However, VAP in ICUs is a complicated and multifaceted clinical disorder that has been linked to high morbidity (4–61%), high mortality (40–43%), and substantial therapeutic costs [1,2,3,4]. The occurrence of VAP exerts a financial burden on the healthcare sector and increases the need for medical supplies [5]. The onset of VAP entails extra health costs ranging from $5000 to $40,000 for each patient and extends hospital stays by 6–30 days [6].

Common risk factors for VAP may include sex, Acute Physiology and Chronic Health Evaluation (APACHE) II score, hypoproteinemia, state of consciousness, prior antibiotic therapy, age, intubation times, mechanical ventilation days, tracheotomy, and underlying diseases such as diabetes and chronic obstructive pulmonary disease [7,8,9,10,11]. Furthermore, previous studies have shown that oral care, bed elevation of 30–45°, and continuing aspiration of subglottic secretions might decrease VAP risk [12,13,14]. The number of elderly patients admitted to ICUs is rising with the aging population. In 1992, 2007, and 2022, the average ages of ICU patients in three extensive studies were 51, 60.7, and 64 years old, respectively [15,16,17]. The incidence of VAP elevates with age. VAP risk increased over 1.15-fold with every 1-year increase, according to Liu et al. [10]. Therefore, it is of great significance to identify high-risk groups regarding VAP, especially in elderly patients, because effective measures can be taken early to reduce the occurrence of VAP.

To the best of our knowledge, there are few validated tools to assess the risk of VAP in elderly patients in the ICU. The nomogram is an intuitive and convenient predictive tool that can quantitatively calculate the hazard ratio of individual clinical events [18,19,20]. It has been widely used in various medical fields [21,22,23]. Consequently, the objectives of this study were to evaluate the significant predictor variables associated with VAP among elderly ICU patients and to construct and validate a nomogram for estimating VAP risk.

## 2. Methods

### 2.1. Study Design and Study Sample

Between January 2016 and March 2021, the data of 293 elderly ICU mechanical ventilation patients from the Wenzhou Hospital of Integrative Medicine were retrospectively reviewed as a training set, and another dataset of 84 elderly ICU mechanical ventilation patients from the same hospital between April 2021 and February 2022 was collected for model validation. If a patient had multiple admissions, only the initial entry was evaluated.

The inclusion criteria included: (1) age 60 years or older; (2) complete clinical data; (3) mechanical ventilation time > 48 h; (4) if multiple VAP infections during hospitalization had occurred, only the first infection was included. We further excluded patients who were admitted to the ICU with a VAP infection. In the end, 137 patients whose mechanical ventilation time did not meet the criteria and 1 patient whose medical record was incomplete were excluded. Finally, 377 patients were included.

Clinical information was collected retrospectively through the real-time hospital infection surveillance system, the software program Zexin (version 3.0), and electronic medical records. This real-time surveillance system could collect data from multiple hospital databases and provide warnings of hospital infection through information such as positive bacterial culture and fever. The cut-off time for the survey was 48 h after extubation in the non-VAP group and the date of infection in the VAP group. The time range for mechanical ventilation days, albumin levels, state of consciousness, intubation times, and tracheotomy/intubation values was from the start of mechanical ventilation to the end of the investigation. The time range for antibiotic use was from admission to the end of the investigation. APACHE II score was taken after the patient was admitted to the ICU.

As a standard rule of thumb, when creating a predictive model for binary or time-to-event outcomes, at minimum, ten events must be collected for each predictor variable (i.e., each β term in the regression equation), then reviewed for inclusion in the predicting model formula; this commonly requires at least ten events per variable (10 EPV) [24,25,26]. In this study, the final model contained five predictors.

**Figure 1:**
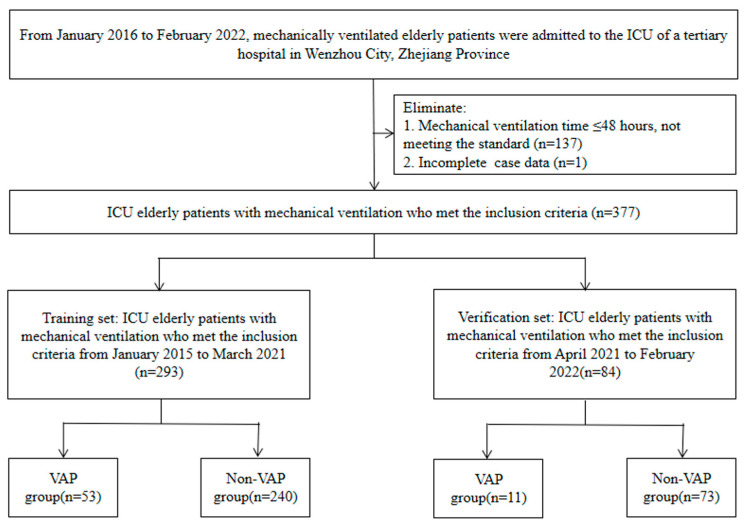
Case screening flow chart.

The screening flow chart is shown in Figure 1. The ethics committee of the Wenzhou Hospital of Integrative Medicine (2022-L079) authorized this research. Due to the retrospective nature of this investigation, the requirement of informed consent was waived by the ethics committee of the Wenzhou Hospital of Integrative Medicine.

### 2.2. Assessment of Potential Predictors

Clinical information, including gender, APACHE II score, hypoproteinemia (defined as a blood albumin concentration below 30 g per liter), long-term combined use of antibiotics, state of consciousness, advanced age, intubation times, mechanical ventilation days, tracheotomy/intubation, and basic disease status (hypertension, diabetes, cerebral infarction, cardiac insufficiency, malignant tumor, Parkinson’s disease, Alzheimer’s disease, hepatic insufficiency, renal insufficiency, chronic obstructive pulmonary disease, and respiratory failure) were collected.

According to clinical significance, the cut-off points of hypoproteinemia, advanced age, and long-term combined use of antibiotics were 30 g/L, ≥80 years old, and ≥2 kinds and ≥7 days of usage. For mechanical ventilation days, APACHE II, and other continuous variables, a two-piece linear regression model with a smoothing function was adopted to evaluate the nonlinear associations of the variable with VAP. A log likelihood ratio test was employed to contrast the one-line linear regression model with a two-piecewise linear model.

### 2.3. Outcome Ascertainment

For the diagnosis of VAP, the clinicians made a preliminary diagnosis, and the hospital’s infection control staff further reviewed it. If there were any disagreements, the two departments would discuss them further to reach a consensus. Based on the 2018 Chinese guidelines for the diagnosis and treatment of hospital-acquired pneumonia and VAP in adults [27], VAP refers to pneumonia arising more than 48 h after tracheotomy or endotracheal intubation for mechanical ventilation. VAP also includes pneumonia that occurs within 48 h after the removal of mechanical ventilation and extubation. A clinical diagnosis of pneumonia can be established via chest X-ray or CT showing new or progressive, infiltrative, solid or ground glass shadows, plus two or more of the following three clinical signs: (1) fever with temperature > 38 degrees; (2) purulent airway discharge; and (3) peripheral blood leukocyte count >10 × 10^9^/L or <4 × 10^9^/L. The VAP concept was consistent with the principles obtained from the Infectious Diseases Society of America and the American Thoracic Society [28].

### 2.4. Statistical Analysis

Continuous variables that were not distributed normally were presented as medians (interquartile range, IQR), and the Mann–Whitney U test was used to compare between-group differences. The categorical variables were presented as *n* (%), and the between-group comparison was made using the chi-square test. Due to the relatively small percentage of missing data (<5%), this study excluded patients with incomplete data. Internal validation was conducted via the repeated self-sampling method (bootstrap of 1000 times), and further validation was conducted with the data of the validation set.

Feature selection and model development. We used the least absolute shrinkage and selection operator (LASSO) regression approach to select the optimum predictors from the training set’s ten potential predictors. Tenfold cross-validation was applied to determine the optimum value of the penalty parameter λ. The selection criterion for the optimal lambda parameter was lambda.1se. (maximum lambda for error mean within one standard deviation of the minimum). Multivariable logistic regression was used to construct a nomogram model using selected variables.

Model performance evaluation. Calibration and discrimination were used to evaluate the nomogram’s performance in predicting VAP risk. The discriminatory ability of the nomogram was evaluated using the receiver operating characteristic (ROC) curve. Generally, an area under the curve (AUC) between 0.71 and 0.90 indicates moderate discrimination, while above 0.90 indicates high discrimination. The calibration capacity of the nomogram was validated utilizing the calibration curve and the Hosmer–Lemeshow goodness-of-fit test, which measures the level of coincidence between the predictive value and the actual estimated values.

Clinical validity evaluation. We conducted a decision curve analysis (DCA) to assess the clinical validity of the nomogram. DCA is a tool that evaluates the net clinical benefit at various threshold probabilities [29,30]. By subtracting the percentage of false-positive patients from the percentage of true-positive patients and weighing the relative harms of not intervening with the negative consequences of unnecessary interventions, the net benefits were calculated [29]. Moreover, the maximum Youden index was used to determine the optimal clinical cutoff value (sensitivity + specificity-1).

Statistical analysis was conducted using R (version 3.4.3) and EmpowerStats (version 3.0, X&Y solutions, Inc., Boston, MA, USA). All statistical tests were two-sided, and a *p*-value < 0.05 was considered as statistically significant.

## 3. Results

### 3.1. Sample Characteristics

A total of 377 elderly ICU patients with mechanical ventilation were included in the current study, and 64 had VAP. VAP was observed in 53 (18.1%) and 11 (13.1%) patients in the training set and the validation set, respectively (Figure 1). Table 1 displays the clinical features of the participants.

### 3.2. Variable Selection

**Figure 2:**
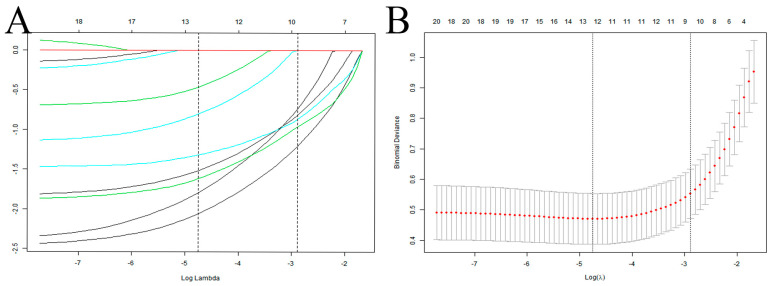
Selection of predictive variables for VAP infection in elderly ICU patients using LASSO regression. (**A**) Profiles of the LASSO coefficients for the ten candidate variables. (**B**) Tenfold cross-validation is used to identify the optimal penalization coefficient (λ) for the LASSO model. Note: The left vertical line indicates the minimum error, while the right vertical line reflects the one standard error of the minimum criteria (1SE criterion).

In this study, 10 variables were dimensionally reduced using LASSO regression, and 5 predictors were selected: hypoproteinemia, long-term combined antibiotics, intubation times, mechanical ventilation days, and tracheotomy/intubation (Figure 2).

### 3.3. Model Development

**Figure 3:**
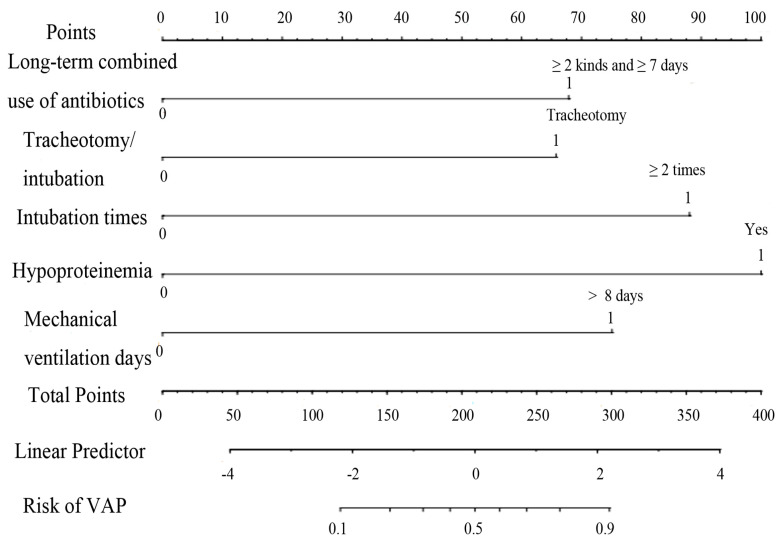
Nomogram of VAP risk prediction for elderly ICU patients with mechanical ventilation.

Hypoproteinemia, long-term combined use of antibiotics, intubation times, mechanical ventilation days, and tracheotomy/intubation were reliable predictive variables of VAP in ICU elderly patients independently (*p* < 0.05) (Table 2), and a nomogram model of VAP in elderly ICU patients was constructed (Figure 3). To make the operation of the nomogram more practical and convenient, we created a web-based online scoring system. Users can open the URL (https://vapmodel.shinyapps.io/dynnomapp/) (accessed on 12 December 2023) when needed and enter variable information or scan a QR code with their cell phone (Figure S1) to obtain the predicted probability of VAP. For example, for an elderly patient who had undergone mechanical ventilation for 12 days, with the combined use of two antibiotics for 10 days, and hypoproteinemia, using the online scoring system indicated that the patient had a VAP risk of approximately 70% (accessed on 6 November 2022) (Figure S2).

### 3.4. Model Performance

**Figure 4:**
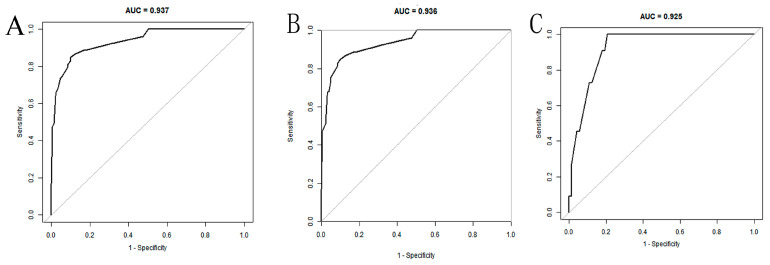
ROC curves of the nomogram: (**A**) training set; (**B**) training set (with bootstrap, 1000 times); (**C**) validation set.

**Figure 5:**
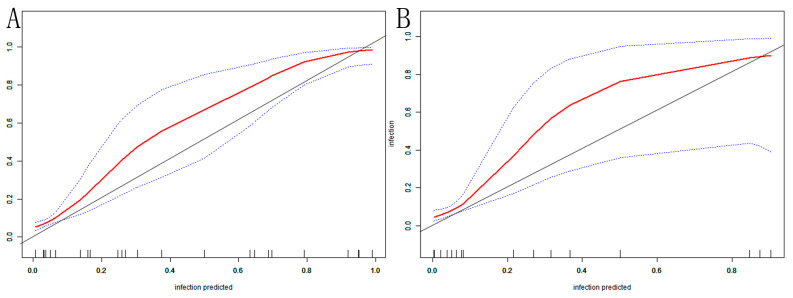
VAP nomogram calibration curve in elderly ICU patients: (**A**) training set; (**B**) validation set. The X-axis depicts the predictive risk of VAP in elderly ICU patients; the Y-axis depicts the actual risk of VAP in elderly ICU patients; the black line depicts the best estimation of the optimal model; the red color is the calibration curve; the blue interval is the 95% CI of the calibration curve.

The AUC of the nomogram was 0.937 (95% CI: 0.902–0.972) in the training set (Figure 4A). At the highest Youden index point, the optimal threshold was 0.207; the corresponding specificity was 90.00%, and the sensitivity was 84.91%. The repeated self-sampling method (bootstrap 1000 times) was used for internal validation of the nomogram, and the AUC was 0.936 (95% CI: 0.901–0.972) (Figure 4B). The AUC was 0.925 (95% CI: 0.867–0.982) in the validation set, indicating that the model had good discrimination (Figure 4C). Hosmer–Lemeshow goodness-of-fit test in the training set *p* = 0.265 > 0.05, validation set *p* = 0.956 > 0.05. The calibration curve and the Hosmer–Lemeshow test showed high consistency with the observed result and the predicted values in the training set (Figure 5A). This was further verified in the validation set (Figure 5B).

### 3.5. Application in Clinical Practice

**Figure 6:**
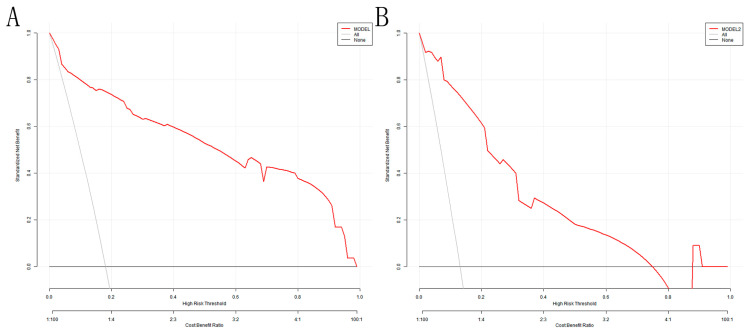
DCA for the nomogram model: (**A**) training set; (**B**) validation set. The Y-axis depicts the net benefit; the gray line in the figure represents the occurrence of VAP in all elderly ICU patients with mechanical ventilation; the black horizontal line indicates that none of the elderly ICU patients developed VAP, and the red represents the decision curve of the risk assessment model.

The DCA analysis shows that the model’s threshold probabilities reached 0–0.98 and 0–0.75 in the training and validation sets, respectively, which could produce net clinical benefits (Figure 6A,B). The optimal threshold of 0.207 for the training set was substituted into Figure 6A, and the clinical net benefit was approximately 75%, indicating a good clinical application value.

## 4. Discussion

In this study, we constructed and validated a nomogram model combining tracheotomy/intubation, intubation times, mechanical ventilation days, long-term combined use of antibiotics, and hypoproteinemia. The area under the curve (AUC) was 0.937 (95% CI: 0.902–0.972) and 0.925 (0.867–0.982) in the training and validation dataset, respectively. The model will help clinicians assess the risk of VAP in elderly ICU patients and identify high-risk patients, which is conducive to taking the necessary interventions to reduce the incidence of VAP early.

Shuhua Li et al. [31] recently established and validated a nomogram model for ventilator-associated pneumonia in elderly ICU patients, but the AUC for the training and validation groups was 0.859 (95% CI: 0.828–0.890) and 0.813 (95% CI: 0.700–0.850), respectively. Additionally, they only included variables with *p* < 0.05 in the univariate analysis in the logistic regression analysis. Zahar et al. [32] developed and validated a VAP model in ICU patients aged > 16 years, The AUC of their model in the training and validation set was 0.881 and 0.848, respectively, but the study did not show the nomogram and calibration curve, and there was no clinical effectiveness evaluation. Wu et al. [33] investigated a VAP prediction model; however, their focus was acute respiratory distress syndrome (ARDS) patients, and they performed a secondary analysis of the early versus delayed enteral nutrition using ARDSNet. In this model, the area under the curve was 0.744, lacking internal and external validation. In elderly ICU patients, few effective tools exist for assessing VAP risk. This research aimed to develop and validate an operable nomogram model.

The predictors included in the nomogram model were tracheotomy/intubation, intubation times, mechanical ventilation days, long-term combined use of antibiotics, and hypoproteinemia. Based on the calibration curve and the AUC results, the nomogram exhibited strong concordance performance as well as good discrimination. Additionally, DCA results in our study demonstrated a good clinical utility of the prediction model. Several studies have proved the advantages of the most recent DCA approach and support its usage [34,35].

In the ICU, the invasive factors of VAP associated with mechanical ventilation include tracheostomy, reintubation, and duration of mechanical ventilation. Tracheotomy impaired the trachea’s normal anatomical and physiological function, corresponding to a previous systematic review performed by Ding et al. [36]. The respiratory system communicates with the external environment immediately, bypassing typical respiratory defense systems, including the oropharynx and cilia; this weakens the cough and reflex role of the trachea, allowing infectious agents to easily colonize the tracheal tube and form biofilm, leading to an increase in the opportunity for VAP infection [37,38,39]. Reintubation was related to the inhalation of potentially pathogenic organisms in the oropharynx [36], and was a risk variable for VAP [40]. The increased number of intubations would damage the airway mucosa and reduce the cilia function, affecting the incidence of VAP. Furthermore, the prevalence of VAP increased from 5% in patients who experienced one day of mechanical ventilation to 65% in patients who experienced 30 days of mechanical ventilation [41]. Moreover, long-term mechanical ventilation would cause damage to the airway mucosa and local cilia function of patients. As mechanical ventilation duration increased, the frequency of procedures, including sputum suction, was elevated, the airway mucosa of patients was damaged more seriously, the cough reflex of patients would be weakened, the barrier function of the respiratory tract was damaged, and the risk of VAP increased. Therefore, reducing invasive operations related to mechanical ventilation, testing the weaning scheme, and minimizing mechanical ventilation time are essential measures to control VAP.

In addition to invasive operations, prior long-term combined use of antibiotics was also a potential independent risk variable for VAP, similar to some prior investigations. The ICU houses the majority of severely ill patients, and the chance of antibiotic administration is extremely high. Antibiotic overuse may modify the infestation of normal microorganisms, resulting in invasion by opportunistic pathogens or drug resistance formation in numerous bacteria strains, hence raising the prevalence of VAP [42,43]. Therefore, it is suggested that care providers follow the principle of antibacterial drug use, carry out a antimicrobial drug susceptibility test before using antibacterial drugs, use antibacterial drugs rationally according to the antimicrobial drug susceptibility test results, and pay attention to the concentration and dosage of antibacterial drugs.

Among patients with VAP, malnutrition plays a key role in addition to medical factors. Albumins are incorporated into all predicting–nutritional variables, and hypoproteinemia is pointed out as a factor of malnutrition, as well as indicating a serious chance of complications [44]. Elderly patients have more underlying diseases than young patients, often have gastrointestinal dysfunction, and are more prone to albumin reduction. Hypoproteinemia may lead to lymphocytopenia, which reduces the immune system’s defensive capacity [45], and in patients with decreased immune resistance, the promotion of the proliferation of drug-resistant bacteria is easy, inducing infection; in particular, severe hypoproteinemia accompanied by pulmonary infection generally had a poor prognosis [46]. The recommendations of the European society for clinical nutrition and metabolism validate the use of nutritional therapy [47]. The variables finally included in this model had clinical significance and a sufficient theoretical basis.

The current study has several advantages. First, the nomogram model was used to establish a diagnostic model for the occurrence of VAP in elderly ICU patients, and a web-based online scoring system was used, which was more intuitive, practical and operable, and conducive to early intervention. Second, a comprehensive evaluation of the model was carried out in all aspects: in addition to the ROC curves showing strong concordance performance, we also used calibration and DCA curves to reflect the model’s calibration degree and clinical validity. Third, internal verification was performed: repeated self-sampling method (bootstrap 1000 times) and verification data divided by the time were used for verification. Temporal validation is a prospective evaluation of a model independent of the modeling data and the modeling process, so it can sometimes be viewed as an external validation [48]. Fourth, it is easy for clinicians to obtain information on the five predictors in our model through electronic medical records. There is no need to increase the patient’s additional examination items or the doctor’s other recording work; these features made our nomogram a useful clinical tool.

However, this study still had limitations. First, this study was a single-center retrospective investigation that excluded a small number of patients with incomplete records, which might be subject to selection bias, and only temporal validation was performed without external validation. Therefore, multicenter prospective studies and external validation should be carried out later. Second, the count of predictors was limited, and only a few readily available and actionable predictors were selected. Although the sample size of this study meets the minimum sample size requirements, the sample sizes of the training set and the verification set were generally small. Third, although the diagnosis of VAP was made by a senior clinician and infection control practitioner to avoid bias caused by misclassification, misdiagnosis and missed diagnosis might still have occurred.

## 5. Conclusions

Our nomogram model showed strong concordance performance, good discrimination, and clinical application value. Using this nomogram model, clinicians can assess VAP risk in elderly ICU patients and identify those at high risk. However, external validation of the nomogram model in larger populations is still required.

## Figures and Tables

**Table 1:** Comparison of general features between the training set and the validation set.

Characteristics	Total (*n* = 377)	Training Set (*n* = 293)	Validation Set (*n* = 84)	*p*-Value
	*N* (%)	*N* (%)	*N* (%)	
**Gender**				**0.532**
Female	124 (32.9%)	94 (32.1%)	30 (35.7%)	
Male	253 (67.1%)	199 (67.9%)	54 (64.3%)	
APACHE II score				0.120
<20 points	130 (34.5%)	107 (36.5%)	23 (27.4%)	
≥20 points	247 (65.5%)	186 (63.5%)	61 (72.6%)	
State of consciousness				0.782
Awake	198 (52.5%)	155 (52.9%)	43 (51.2%)	
Comatose	179 (47.5%)	138 (47.1%)	41 (48.8%)	
Hypoproteinemia				0.067
No	247 (65.5%)	199 (67.9%)	48 (57.1%)	
Yes	130 (34.5%)	94 (32.1%)	36 (42.9%)	
Long-term combined use of antibiotics				0.181
≥2 kinds and ≥7 days	68 (18.0%)	57 (19.5%)	11 (13.1%)	
Other situations	309 (82.0%)	236 (80.5%)	73 (86.9%)	
Advanced age				0.805
<80 years old	220 (58.4%)	170 (58.0%)	50 (59.5%)	
≥80 years old	157 (41.6%)	123 (42.0%)	34 (40.5%)	
Intubation times				0.562
1 time	355 (94.2%)	277 (94.5%)	78 (92.9%)	
≥2 times	22 (5.8%)	16 (5.5%)	6 (7.1%)	
Mechanical ventilation days				0.671
≤8 days	223 (59.2%)	175 (59.7%)	48 (57.1%)	
>8 days	154 (40.8%)	118 (40.3%)	36 (42.9%)	
Tracheotomy/intubation				0.052
Intubation	290 (76.9%)	232 (79.2%)	58 (69.0%)	
Tracheotomy	87 (23.1%)	61 (20.8%)	26 (31.0%)	
Number of basic diseases				0.226
≤2	189 (50.1%)	142 (48.5%)	47 (56.0%)	
>2	188 (49.9%)	151 (51.5%)	37 (44.0%)	

**Table 2:** Multivariate logistic analysis of VAP infection in ICU elderly patients.

Variables	OR	95% CI	*p*-Value
Hypoproteinemia	11.516	4.384–30.248	<0.001
Intubation times	8.598	1.618–45.685	0.012
Tracheotomy/intubation	4.986	1.880–13.219	0.001
Mechanical ventilation days	6.267	2.194–17.899	<0.001
Long-term combined use of antibiotics	5.249	1.938–14.221	0.001

## Data Availability

The datasets generated and/or analyzed during the current study are not publicly available due to confidentiality requirements but are available from the corresponding author on reasonable request.

## Supplementary Materials

The following supporting information can be downloaded at: https://www.mdpi.com/article/10.3390/arm92010010/s1, Figure S1; Figure S2.
