# Supplementary figures and images for: Constructing a Nomogram Model to Estimate the Risk of Ventilator-Associated Pneumonia for Elderly Patients in the Intensive Care Unit
Source: Adv Respir Med. 2024 Jan 19;92(1):77–88. doi: 10.3390/arm92010010 (PMC10885902; doi:10.3390/arm92010010)

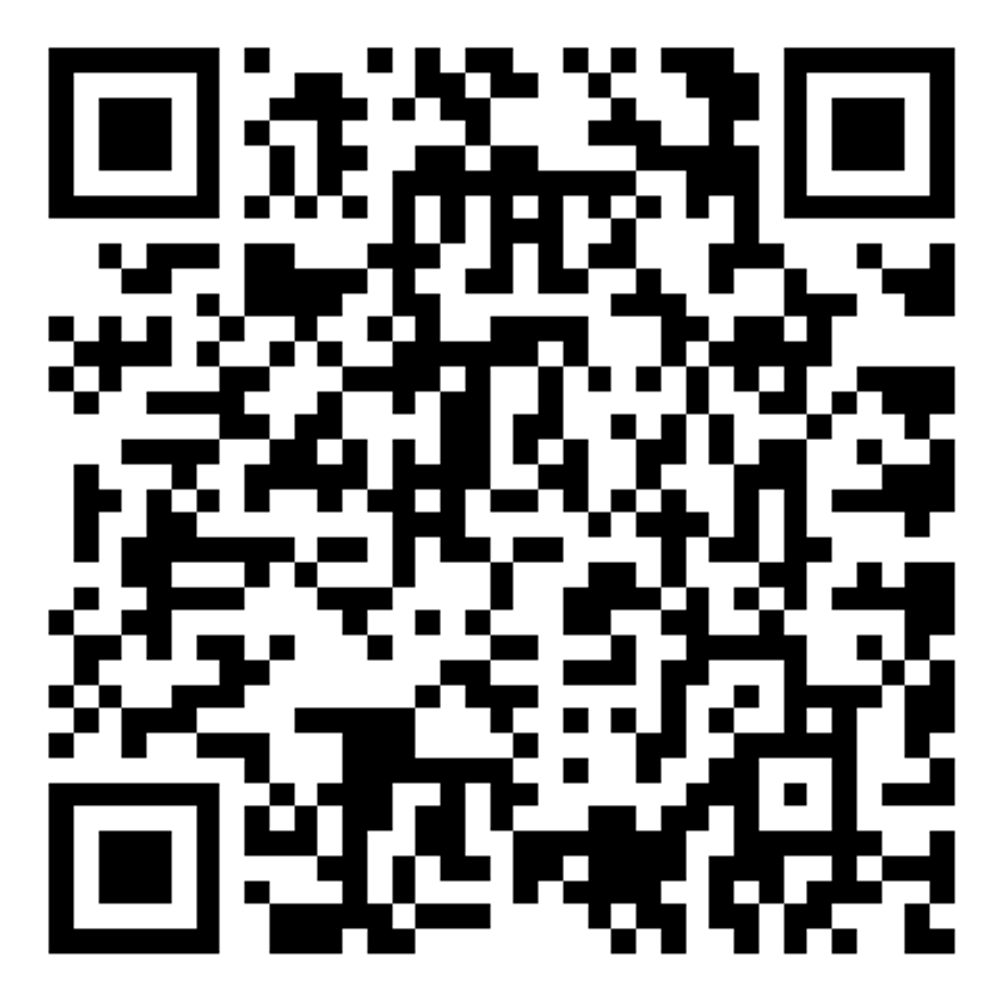

Supplement: Supplementary file 1 [file arm-92-00010-s001.zip › supplementary chart/Figure S1.tif]

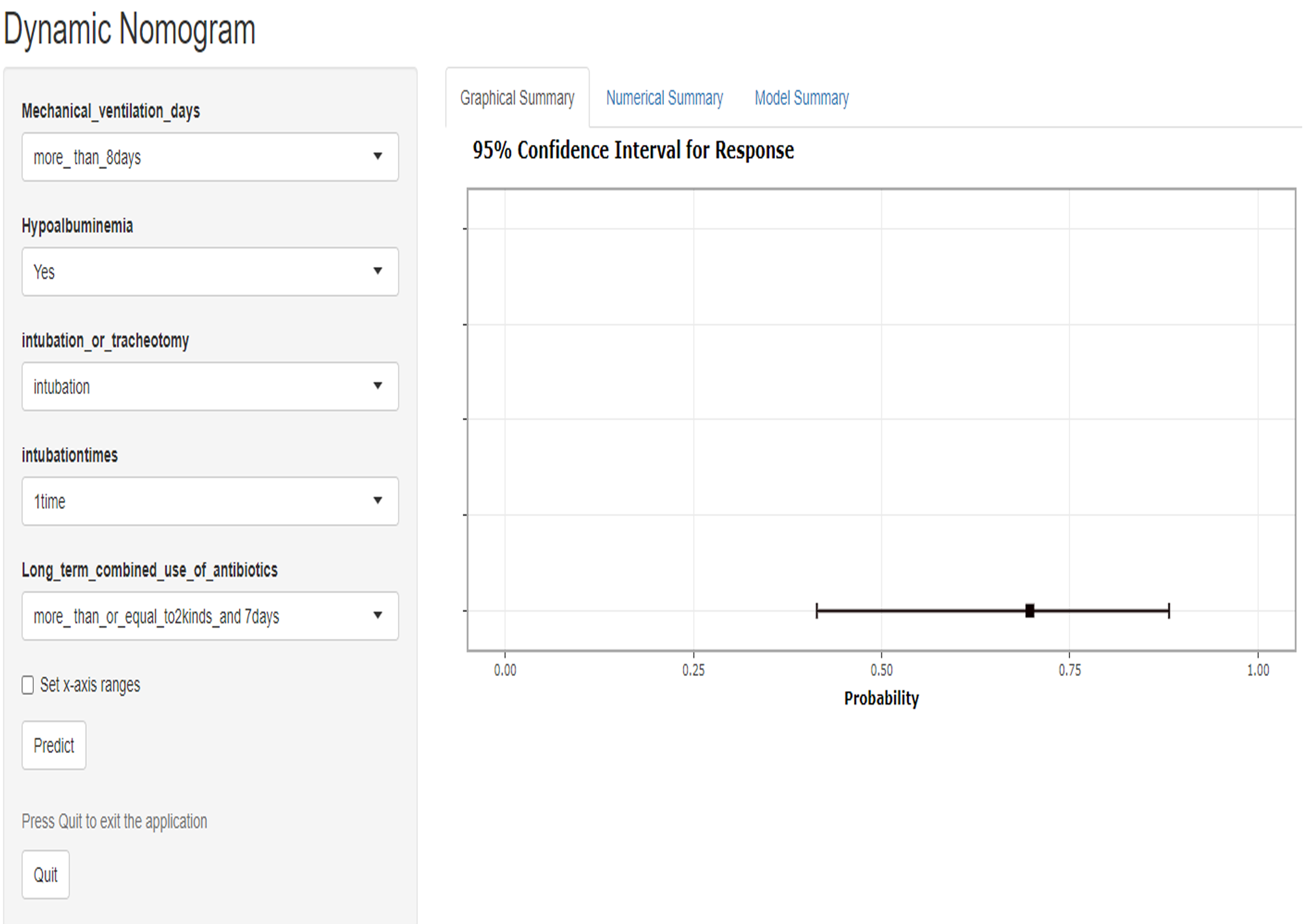

Supplement: Supplementary file 1 [file arm-92-00010-s001.zip › supplementary chart/Figure S2.tif]
